# 116 Expert Agreement on definitions for compensatory protective step strategies: Delphi method study

**DOI:** 10.1093/eurpub/ckae114.168

**Published:** 2024-09-26

**Authors:** Maria Melo-Alonso, Alvaro Murillo-Garcia, Carmen Padilla-Moledo, Santos Villafaina, Maria Carmen Gomez-Alvaro, Felipe Alejandro Morcillo-Parras, Juan Luis Leon-Llamas, Narcis Gusi

**Affiliations:** Faculty of Sport Sciences, University Of Extremadura, Caceres, Spain; Institute of Research and Innovation in Sport Sciences, Caceres, Spain; Faculty of Sport Sciences, University Of Extremadura, Caceres, Spain; Institute of Research and Innovation in Sport Sciences, Caceres, Spain; Faculty of Education, University of Cadiz, Cadiz, Spain; Faculty of Sport Sciences, University Of Extremadura, Caceres, Spain; Institute of Research and Innovation in Sport Sciences, Caceres, Spain; Faculty of Sport Sciences, University Of Extremadura, Caceres, Spain; Institute of Research and Innovation in Sport Sciences, Caceres, Spain; Faculty of Sport Sciences, University Of Extremadura, Caceres, Spain; Institute of Research and Innovation in Sport Sciences, Caceres, Spain; Faculty of Sport Sciences, University Of Extremadura, Caceres, Spain; Institute of Research and Innovation in Sport Sciences, Caceres, Spain

## Abstract

**Purpose:**

The older adults have a high probability of suffering an unexpected fall during activities of daily living, this can generate fear of fall, changes in gait patterns, decreased mobility, reduced social contact and impaired ability to perform different activities. Previous studies of gait in the presence of a perturbance identified and analyzed several protective gait strategies to prevent a fall. But there was a lack of common wording and definitions for the same strategy that limits the comparison and scientific synergic advance among researchers and professionals.

**Objective:**

to elaborate and clarify the definitions of compensatory protective step strategies to create a single common terminology to be used by all related-professionals (e.g., physical educators, physiotherapists, occupational therapists, doctors and researchers).

**Methods:**

the study followed the Conducting and Reporting of Delphi Studies guideline (CREDES) and a chronological order 1) review of the literature (scoping review), 2) step-by-step creating a quantitative questionnaire (Likert-type scale from 1 to 5) and qualitative assessment of degree the pertinence, wording and identification of each of the definitions (items=14), 3) same number of experts selection from different professional backgrounds (N = 14), 4) Aike's V coefficient was used to determine content validity.

**Results:**

showed demanding levels of validity (V ≥ 0.68) and none of the strategy definitions were eliminated, all of them passed Aiken’s V critical value. Conclusion: the definitions of 14 compensatory protective step strategies (13 strategies mentioned in the scoping review and one added by the panel of experts) have been elaborated and clarified. The limb collisions were not included by the consensus of the panel of experts because it was considered as an isolated action and information about its protective function is lacking. These identifications could be used as innovative specific targets to include in health enhancing physical activity programs

**Funding:**

The Spanish National R + D + i Plan co-funded by the Spanish Ministry of Sciences and Innovation (reference PID2019-107191RB I00/AEI/10.13039/501100011033). The author J.L.L.-L. was supported by a grant from the Spanish Ministry of Education, Culture and Sport (FPU18/05655).

